# High frequency ultrasound for pyoderma gangrenosum: A case series

**DOI:** 10.1016/j.jdcr.2025.05.008

**Published:** 2025-06-04

**Authors:** Katelin R. Ross, Shivani Thacker, Mekaila Hudson, Moira Shea, Alex G. Ortega-Loayza

**Affiliations:** Department of Dermatology, Oregon Health & Science University, Portland, Oregon

**Keywords:** diagnosis, high frequency ultrasound, imaging, inflammatory conditions, neutrophilic dermatoses, pyoderma gangrenosum, ulcers, wound healing

## Introduction

Pyoderma gangrenosum (PG) is a rare neutrophilic dermatosis that presents with chronic, painful, inflammatory lesions.^1^ The most common subtype, ulcerative PG, is characterized by pustules that rapidly expand into skin ulcers with raised, violaceous, and undermined borders.^1^ PG is difficult to diagnose due to overlapping clinical features with other ulcerative skin conditions and nonspecific histologic findings.^1^ Despite development of validated scoring criteria, such as PARCELSUS (a diagnostic framework for PG that stands for progression of disease, assessment of relevant differential diagnoses, reddish-violaceous wound border, amelioration by immunosuppressive drugs, characteristically irregular ulcer shape, extreme pain, localization of lesion at site of trauma, suppurative inflammation on histopathology, undermined wound border, systemic disease associated), Delphi, and Su, it still remains a diagnostic challenge.^2^^,^^3^ Misdiagnosis is not uncommon, leading to delays in treatment, unnecessary intravenous antibiotics, surgical procedures, and even amputations.^4^ Recently, noninvasive diagnostic tools have gained traction when assessing patients with ulcerations.^5^ High frequency ultrasound (HFUS) is a rapidly expanding modality in dermatology due to its high-resolution imaging capabilities that discern subtle differences in tissues. HFUS can dynamically characterize lesion extent, depth, and vascularity; however, few studies have investigated its utility in skin ulcers.^5^^,^^6^ In this report, we highlight the use of HFUS using a GE Linear 6-24 MHz transducer to distinguish phases of PG and its potential to differentiate PG from a clinical mimicker.

## Case 1

**Fig 1:**
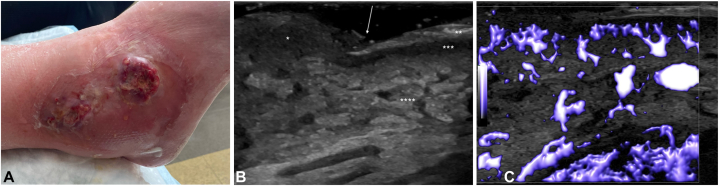
**A,** Recurrent PG ulceration on right external ankle. **B,** HFUS with hypoechoic wound (∗) bulging up through skin; hyperechoic epidermis (∗∗) and hypoechoic dermis (∗∗∗) with abrupt disruption of tissue layers at the ulcer edge (*arrow*); cobblestoning (interstitial edema) in the subcutaneous layer (∗∗∗∗). **C,** HFUS microvascular imaging showing highly vascular wound bed with acute vessel angle branching. *HFUS*, High frequency ultrasound; *PG*, pyoderma gangrenosum.

A 75-year-old woman with history of PG presented with 2 weeks of a rapidly progressive ulcer on her right lateral ankle. Her PARACELSUS score was 11, confirming the diagnosis of recurrent PG (Fig 1, *A*). The ulcer was imaged using HFUS which demonstrated abrupt discontinuation of epidermis and dermis where the wound bulged through the skin. With HFUS, normal epidermis is seen as a superficial hyperechoic layer due to the high keratin content, and dermis a slightly more hypoechoic layer due to high collagen content. In this case, the decreased echogenicity of the dermis layer was exaggerated by inflammation, seen as a hypoechoic layer below the epidermis adjacent to the wound. There was significant interstitial edema creating a “cobblestone” appearance in the subcutaneous layer, with interdigitating hypoechoic regions surrounding hyperechoic fat lobules akin to a cobblestone street (Fig 1, *B*). Tissue vascularity was imaged using microvascular imaging software (similar to power Doppler to detect slow flow) which demonstrated increased blood flow in the subcutaneous layer (Fig 1, *C*). The loss of normal tissue architecture and increased vascularity are ultrasound findings that suggest an acute inflammatory process, and are appreciated in conditions such as cellulitis.^5^

## Case 2

**Fig 2:**
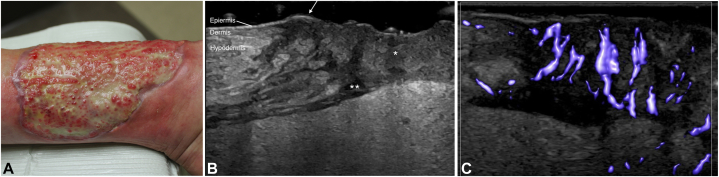
**A,** Healing PG ulceration on left lower extremity. **B,** HFUS of edge of ulcer. There is a hypoechoic wound bed (∗) with an edematous drainage tract deep to the ulcer (∗∗). At the wound edge, there is normal hyperechoic epidermis, slightly hypoechoic dermis, and subcutaneous layer without evidence of interstitial edema. The thin hyperehcoic epidermis extends from normal tissue at the ulcer edge over the wound bed (*arrow*). **C,** HFUS microvascular imaging shows moderate vascularity with organized, linear pattern in wound bed. *HFUS*, High frequency ultrasound; *PG*, pyoderma gangrenosum.

A 57-year-old woman presented for follow-up with a 16.5-month history of a PG ulcer on her left lower extremity while undergoing immunosuppressive therapy. At initial presentation, her PARCELSUS score was 12 (Fig 2, *A*). She showed evidence of epithelial growth between the ulcer bed and surrounding skin indicative of Gulliver’s sign—a clinical clue for the initiation of the healing phase of PG.^7^ HFUS revealed a hypoechoic wound bed with well-defined skin layers at the perimeter. There was a hypoechoic drainage tract deep to the wound. The echogenic epidermis extended continuously from the healthy wound edge over the ulcer, corresponding clinically with Gulliver’s sign. The dermis and subcutaneous layers surrounding the ulcer were without evidence of interstitial edema and showed normal tissue architecture (Fig 2, *B*). Microvascular color flow showed modest vasculature without significant vessel branching (Fig 2, *C*). Together, these findings suggest tissue reorganization and healing.

## Case 3

**Fig 3:**
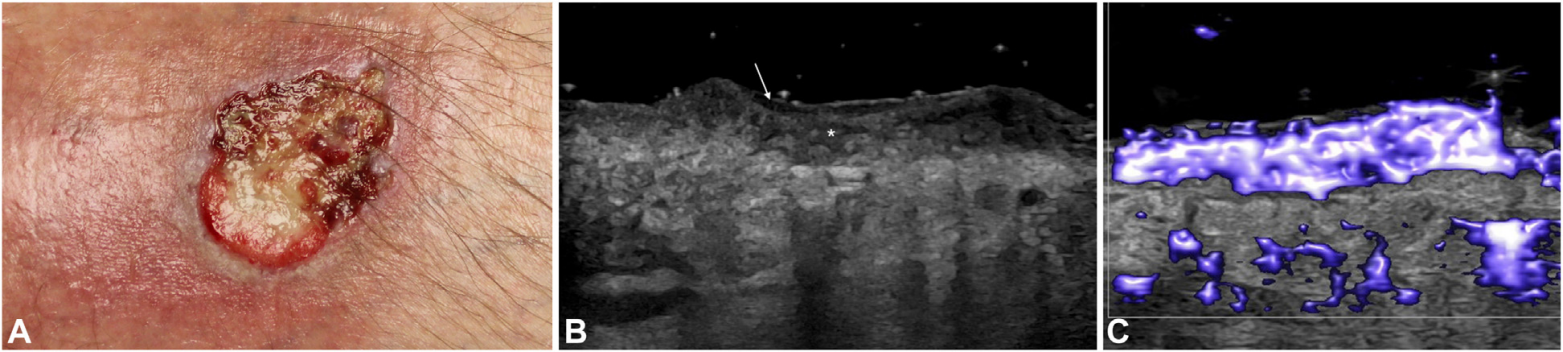
**A,** Basal cell carcinoma (PG-mimicking ulceration) on right lower extremity. **B,** HFUS demonstrates shallow heterogenous wound bed (∗) and hyperehoic epidermis seen over entire wound with underlying hypoechoic layer (*arrow*). **C,** HFUS microvascular imaging with extensive hyperemia in wound bed involving epidermis layer. *HFUS*, High frequency ultrasound; *PG*, pyoderma gangrenosum.

A 60-year-old man was referred to our clinic for possible diagnosis of PG. He had a large, progressive nonhealing skin ulcer on his right lower extremity that started after an injury 2 years prior. His PARCELSUS score was 10, suggestive of possible PG (Fig 3, *A*). HFUS was used to image the ulcer. In contrast to the HFUS findings of PG, ultrasound showed an intact epidermis layer with a hypoechoic layer just below, a finding not seen in the PG ulcers. Additionally, it exhibited extensive hyperemia throughout the lesion involving the dermis and epidermis layers (Fig 3, *B* and *C*). A skin biopsy was performed and revealed aggregates of basaloid epithelial cells characterized by hyperchromatic nuclei and scant cytoplasm, with peripheral nuclear palisading, consistent with nodular and fibrosing basal cell carcinoma. This patient was appropriately scheduled for surgical intervention.

## Discussion

The diagnosis of PG remains a clinical challenge due to the lack of unique specific markers. Histological findings are often useful in ruling out clinical mimickers; however, the classic diffuse neutrophilic infiltration associated with PG ulcerations is typically observed only in the acute phase and is not exclusive to PG. In this this case series, we highlight the potential benefits of incorporating HFUS in the diagnosis of PG.

According to diagnostic criteria proposed in 2019, a PARCELSUS score of 10 or greater indicates a high likelihood of PG with high sensitivity and low specificity.^8^ Incorporating HFUS into the workup in these cases provided additional data to help discern the phases of PG and guide therapy. Novel findings demonstrated abrupt epidermis and dermis disruption in active PG and tissue reorganization and regrowth in healing PG. With HFUS, we imaged Gulliver’s sign as an echogenic layer extending above the underlying wound bed demonstrating the regrowing superficial epithelium over the lesion.

Additionally, in our third case, PARCELSUS score indicated a high likelihood of PG; however, HFUS revealed a significant increase in vascularity compared to PG lesions. These findings were consistent with basal cell carcinoma as described in previous reports.^9^ Overall, HFUS revealed aspects of wound extent that could not be appreciated on clinical exam alone in all 3 cases.

The current diagnostic frameworks for PG rely on subjective assessments, making them prone to inconsistency and misinterpretation, particularly for clinicians less familiar with PG.^10^ This results in increased healthcare utilization (eg. unnecessary use of intravenous antibiotics, surgical debridement, amputations) and limitations in research endeavors.^11^^,^^12^ The identification and validation of objective signs that can be used alone or incorporated within these diagnostic criteria would optimize the diagnosis of PG and potentially guide management. These case examples emphasize the practical integration of HFUS in clinical practice as a method for point of care assessment.

Advancing technology and validated research methods improve diagnostic accuracy and reduce patient harm. HFUS is a noninvasive tool that can aid in PG identification and characterization, providing confirmation of diagnosis and the possibility of avoiding skin biopsy. It offers an opportunity for future reverse translational research, lending further insight in the understanding of PG with validation of HFUS as a part of the diagnostic framework.

## Conflicts of interest

Dr Ortega-Loayza is the former President of the Pacific Dermatology Association and serves as an associate editor for *Dermatology (Karger)* and editorial board member of the *American Journal of Clinical Dermatology*. In addition, he is a consultant for Genentech and Guidepoint and an advisor to Bristol Meyer Squibb, Boehringer Ingelheim, and Janssen. He has received research grants from the Lilly, Janssen, Incyte, and Pfizer. He is supported by National Institutes of Health National Institute of Arthritis and Musculoskeletal and Skin Diseases R01 AR083110. Authors Ross, Hudson, Shea, and Dr Thacker have no conflicts of interest to declare.
